# Deficient perceptions and practices concerning elevated lipoprotein(a) among specialists in Singapore

**DOI:** 10.3389/fcvm.2025.1527351

**Published:** 2025-02-14

**Authors:** Wann Jia Loh, Jing Pang, Oliver Simon, Dick C. Chan, Gerald F. Watts

**Affiliations:** ^1^ Medical School, University of Western Australia, Perth, WA, Australia; ^2^ Department of Endocrinology, Changi General Hospital, Singapore, Singapore; ^3^ Medical Affairs Department, Duke-NUS Medical School, Singapore, Singapore; ^4^ Medical Affairs Department, Novartis (Singapore) Pte Ltd, Singapore, Singapore; ^5^ Department of Cardiology and Internal Medicine, Royal Perth Hospital, Perth, WA, Australia

**Keywords:** lipoprotein(a), Lp(a), detection lipoprotein a, cascade testing, opportunistic testing, Lp(a) questionnaire, knowledge, practice, awareness Lp(a)

## Abstract

**Background:**

Multiple guidelines recommend detection of and early risk factor management for elevated lipoprotein(a) [Lp(a)]. Effective implementation requires assessment of knowledge and practices regarding elevated Lp(a) among medical specialists.

**Aim:**

To assess awareness, knowledge and practices of the detection and treatment of elevated Lp(a) among specialist physicians in Singapore.

**Methods:**

Seventy-five practicing specialists in cardiology (*n* = 33) or endocrinology (*n* = 42) anonymously completed a structured questionnaire that assessed the above three aims.

**Results:**

The majority of respondents (83%) rated their familiarity with Lp(a) as at least average, with a greater percentage of endocrinologists being less familiar with Lp(a) than cardiologists (29% vs. 3%, *P* < 0.01). 57% were aware of at least one guideline or consensus statement on Lp(a), which was more frequent among cardiologists than endocrinologists (70% vs. 48%, *P* = 0.05). There were major gaps in knowledge of the prevalence, pathophysiological role, clinical significance and management of elevated Lp(a), correct responses being less than 30%; 44% of respondents (33% cardiologists and 52% endocrinologists) never tested for Lp(a), lack of effective treatment being the most common barrier (59%). A higher proportion of specialists that did not test for Lp(a) rated familiarity with Lp(a) as being low compared with specialists that tested for Lp(a) regularly (33% vs. 13%, *P* = 0.02). Education and training were considered most useful for improving care of patients with elevated Lp(a).

**Conclusion:**

Major gaps in awareness, knowledge and management of elevated Lp(a) were identified among specialists in Singapore. Education and training of specialists are required to overcome initial barriers to testing.

## Introduction

Elevated lipoprotein(a) [Lp(a)] is a co-dominantly inherited hypercholesterolaemia which is universally acknowledged as an independent risk factor for atherosclerotic cardiovascular disease (ASCVD) and calcific aortic valve stenosis (CAVS) (1–7). The prevalence of elevated Lp(a) ranges from 10% to 30%, depending on ethnicity, making elevated Lp(a) an important public health issue (8). Recent international guidelines recommend co-ordinated action to identify and manage patients with elevated Lp(a) (1–5, 7, 9). However, elevated Lp(a) remains under-recognized and under-detected (3, 10–14).

We previously found that Lp(a) is a predictor of coronary artery disease (CAD) in a multi-ethnic Singaporean population (15). However, the majority of people with elevated Lp(a) in Singapore remain undetected, including those with clinical ASCVD (10). Implementation of guidelines requires an assessment of current knowledge and practices regarding elevated Lp(a) (1). We aimed to investigate awareness, knowledge and management of elevated Lp(a) among cardiologists and endocrinologists in Singapore.

## Methods

Practicing specialists in cardiology or endocrinology in the public and private health sector in hospitals and clinics throughout Singapore were invited to participate in an anonymised study. We aimed to have a total of 75 respondents, which was about one-sixth of registered consultant specialists in cardiology and endocrinology, particularly targeting responses from doctors who were actively practicing. A structured questionnaire of 30 multiple choice questions on Lp(a) was developed based on expert recommendations and guidelines on Lp(a) by the authors (WJL and JP) in collaboration with a lipid specialist (GW) (1, 16). To avoid bias, the acquisition of responses from study participants was conducted by a third-party company (Ipsos Pte Ltd, Singapore) over a period of 1.5 month from April 2024. The authors were blinded to the identity and responses of the study participants. 50 specialists (25 from each discipline) completed the questionnaire in-person, and another 25 (8 cardiologists and 17 endocrinologists) completed the questionnaire after online invitation, while 60 specialists who were approached declined. Each study participant received a small monetary compensation for their time.

The survey inquired about the following aspects of Lp(a): awareness (familiarity with the condition, guidelines, clinical significance), knowledge (molecular structure, metabolism, physiological function, prevalence, and biological variability), management practices (risk stratification, treatments) and opinions on testing and detection. A 7-point Likert scale was used for the question on familiarity with Lp(a); Score 1–2 was categorised as “not familiar”, score 3–5 was categorised as 'somewhat familiar’ and score 6–7 was categorised as “very familiar”. De-identified demographic data including sex and years of practice were recorded. This survey was part of a series of quality improvement projects to improve the detection and management of Lp(a) locally. The project did not involve personal health information of human subjects from study participants and ethical board review was not required according to institutional guidelines. Responses to individual questions were presented as proportions (percentage). Statistical analyses of categorical variables were performed using chi-square tests, with *P* value <0.05 considered statistically significant.

## Results

Of 75 respondents, 71% were working in the public health sector (70% in cardiology, 71% in endocrinology) with the remainder 29% were practicing in the private health sector (30% in cardiology and 29% in endocrinology). 61% were male and all spent an average of 88% of their work time in patient care. The mean time in clinical practice after completion of specialist training was 13.7 years (range 1–29 years). The mean number of patients reviewed in their clinics per month was 240 patients (range 40–600 patients), with >90% having ASCVD or being at high risk of ASCVD.

**Figure 1:**
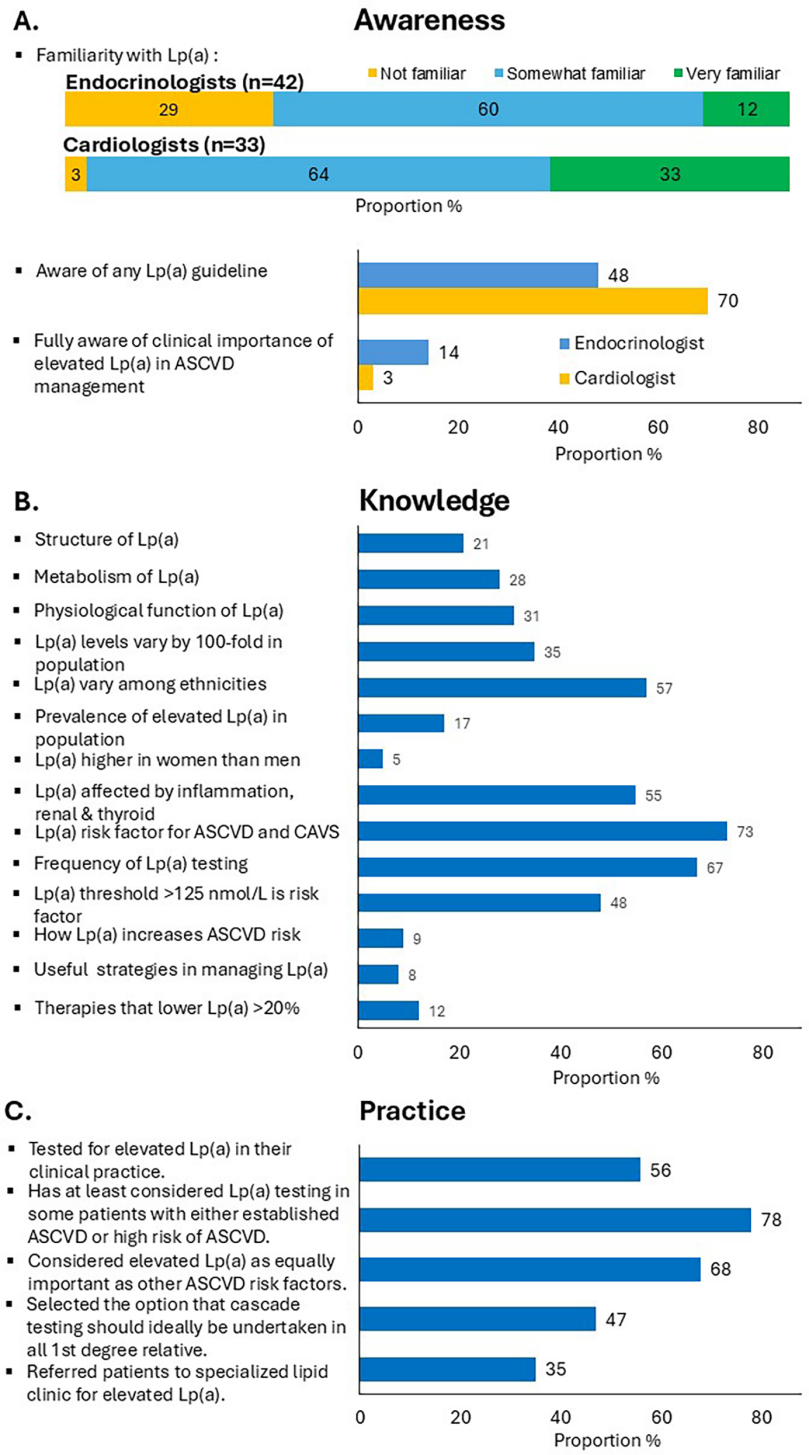
Proportion of specialists responding to specific questions on **(A)** awareness, **(B)** knowledge, and **(C)** clinical practice concerning Lp(a) using the study questionnaire.

Figure 1 summarises the survey results across awareness, knowledge and management practices of elevated Lp(a). The majority of respondents (83%) rated their familiarity with Lp(a) as 'somewhat familiar’ or “very familiar”. Endocrinologists were less familiar with Lp(a) than cardiologists (29% vs. 3%, *P* < 0.01). 57% responded that they were aware of at least one guideline or consensus statement on Lp(a), with a greater percentage of cardiologists than endocrinologists (70% vs. 48%, *P* = 0.05). However, only 9% correctly identified the importance of measuring Lp(a) for managing ASCVD risk (14% of endocrinologists and 3% of cardiologists, *P* = 0.09). The proportion of respondents correctly identifying the molecular structure, metabolism and physiological function of Lp(a) were only 21%, 28% and 31%, respectively. Just over half correctly identified that Lp(a) levels varied with ethnicity (57%) and were affected by medical conditions, such as inflammatory, renal and thyroid disorders (55%). However, only one-fifth selected the correct range for Lp(a) variability among individuals (35%) and the correct prevalence of elevated Lp(a) in the general population (17%). Only 5% of the respondents correctly identified that Lp(a) concentrations were higher in women than in men. A high proportion of respondents correctly identified that elevated Lp(a) was a potent risk factor for ASCVD and CAVS (73%), with testing for Lp(a) required only once in most people, except when secondary causes are suspected or treatment initiated (67%). 48% selected the correct threshold above which cardiovascular risk was increased [Lp(a) level of 125 nmol/L or 50 mg/dl] (1). However, few respondents correctly identified the role of Lp(a) in atherosclerosis (9%), and useful strategies for managing elevated Lp(a) (8%), including lipid-lowering therapies that could lower Lp(a) by more than 20% (12%). Overall, there were no major differences in knowledge of Lp(a) between the two specialties.

68% of respondents considered elevated Lp(a) as an equally important risk factor for assessing patients with premature ASCVD compared with other traditional ASCVD risk factors (73% of cardiologists, 64% endocrinologists). However, almost half the participants indicated that they never requested a test for Lp(a) (44%) (33% of cardiologists, 52% endocrinologists, *P* = 0.09). Only 5 specialists (6.7%) tested for Lp(a) in at least 80% of their patients with ASCVD. A higher proportion of specialists that did not test for Lp(a) rated their familiarity with Lp(a) as low, compared with specialists that regularly test for Lp(a) (33% vs. 13%, *P* = 0.02). However, there was no statistical difference in awareness of Lp(a) guidelines (*P* = 0.67), gender, or years of specialist experience (more vs. less than 10 years) among specialists that test vs. those that did not test for Lp(a). 78% reported that they would have at least considered Lp(a) testing in some patients with either established ASCVD or at high risk of ASCVD. Among specialists who test for Lp(a), 38% have never offered cascade testing. However, 47% of all respondents replied that cascade testing should ideally be considered in first-degree family members of patients with elevated Lp(a), Figure 1C.

**Figure 2:**
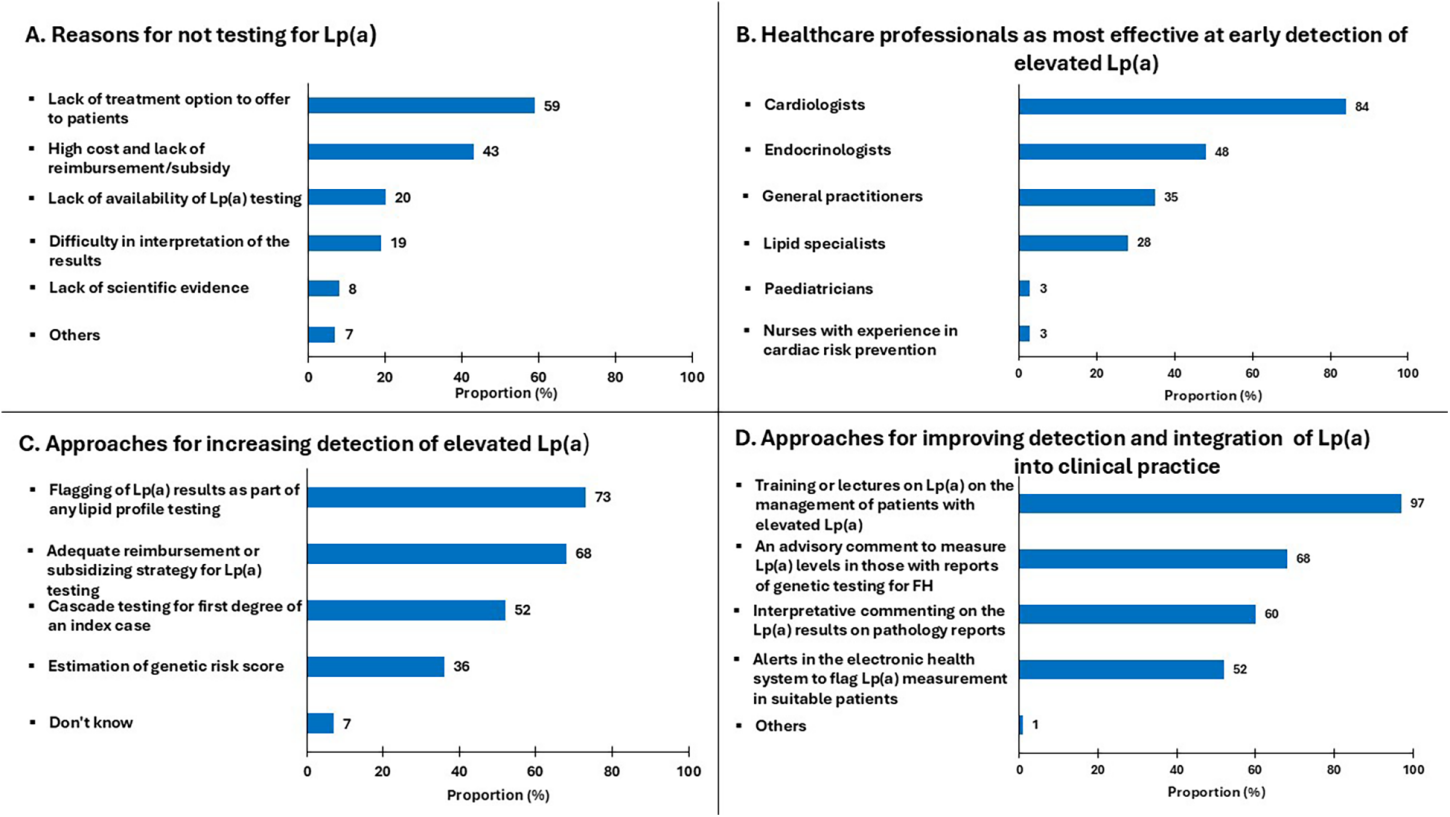
Proportion of specialists who responded to questions regarding: **(A)** reasons for not testing Lp(a); **(B)** healthcare professionals most effective at early detection of elevated Lp(a); **(C)** approaches for increasing detection of elevated Lp(a); **(D)** approaches for improving detection and integration of Lp(a) into practice.

Figure 2 summarises the barriers to Lp(a) testing and opinions on improving the implementation of the use of Lp(a) in clinical practice. The most common barriers to testing for Lp(a) was the lack of effective-Lp(a) lowering therapies (59%), followed by the high cost and lack of reimbursement or subsidy for testing (43%) (Figure 2A). Approximately 20% of respondents considered poor access to Lp(a) assays and difficulty in the interpretation of results as barriers to testing for Lp(a). A similarly low proportion of cardiologists and endocrinologist (8%) were not convinced of the additional cardiovascular risk due to Lp(a) on the basis of inadequate scientific evidence.

As shown in Figure 2B, cardiologists were considered the most appropriate healthcare professionals for the early detection of elevated Lp(a) (84%). This was followed by endocrinologists (48%), general practitioners (35%), lipid specialists (28%), paediatricians (3%) and nurses with experience in cardiovascular prevention (3%). More than half of specialists considered adequate reimbursement/subsidy for opportunistic testing (68%), and cascade testing (52%) as important enabling strategies, 36% also suggesting that estimation of *LPA* genetic risk score may be helpful in detecting elevated Lp(a). There were comparable responses from both specialties on how to improve awareness and integration of Lp(a) in clinical practice. Almost all responders identified that training and education on Lp(a) management was essential (97%) and more than half also referred to other approaches for improving the detection of Lp(a); this includes an advisory comment on laboratory reports to measure Lp(a) in patients suspected of having familial hypercholesterolemia [FH] (68%), interpretive comments on Lp(a) results issued by laboratories (60%), and alerts in electronic health systems to screen high-risk patients for Lp(a) (52%) (Figure 2D).

## Discussion

This is the first inquiry in the Asia-Pacific region on knowledge, awareness and practices concerning Lp(a) among medical specialists. Among practicing cardiologist and endocrinologists in Singapore, we identified major gaps in awareness, knowledge and management as well as barriers in detection, adding to the limited literature (17, 18).

Since cardiology and endocrinology are among the forefront disciplines managing patients at high and very high risk of ASCVD, their perceptions and opinions of this public health problem are critical. Although the majority of respondents considered elevated Lp(a) to be an equally important risk factor for ASCVD, the value of testing was under-recognised as 44% of specialists did not test for Lp(a). We found that lower familiarity with Lp(a) was associated with higher likelihood of non-testing of Lp(a). Of note, there was a higher proportion of endocrinologists than cardiologists that rated their familiarity to be low and this corresponded with a lower Lp(a) testing rate. We also found that almost all specialists considered education and training programs for doctors to be most useful for improving the care of patients with elevated Lp(a).

Collectively, the gaps in knowledge and management practices suggested a lack of translation of the key messages in the Lp(a) guidelines to clinical practice, such as in the threshold of Lp(a) and management strategies. Additionally, this was reflected by the lack of awareness of expert recommendations that state that elevated Lp(a) should be employed as a risk-enhancing factor to promote intensification of other cardiovascular risk factors to lower the overall cardiovascular risk (1, 5). Another perceived barrier was the high cost of Lp(a) testing, but the cost of Lp(a) at the time of writing was SGD$70–$100 (US $50–$75) in the private sector, and often lower than SGD $40 (USD $30) per test in some restructured hospitals depending on the patient's subsidy programme, which was cheaper than renal, liver or thyroid panels in some medical institutes (information gathered from author's work experience and personal contacts). Our data revealed a lower testing rate compared with a survey conducted in the European lipid clinic network (75.5% of the 151 specialists) but higher than a study from Pennsylvania, USA (31% of 126 doctors) (17, 18). Similar to our study, the common barriers identified were lack of reimbursement, lack of availability of Lp(a) test in their centres, and the perceived notion of lack of management for elevated Lp(a) (17, 18). The lack of testing and awareness is of concern, because elevated Lp(a) is common in lipid or specialist clinics (19).

Important detection strategies to identify patients with elevated Lp(a) at high-risk for ASCVD include systematic testing of patients at high risk of ASCVD including FH and cascade testing of their family members (3, 20, 21). More than a half of our respondents considered that cascade testing for first-degree of an individual with elevated Lp(a) could be employed to increase the detection of elevated Lp(a), although this was generally not being practiced. The effectiveness of cascade testing in detecting elevated Lp(a) is supported by our previous studies demonstrating that cascade testing of families (adults or children/adolescents) for elevated Lp(a) from affected probands can, on average, identify one new case of elevated Lp(a) per two family members tested (20, 21). However, the cost effectiveness of cascade testing of elevated Lp(a) requires evaluation.

Our results demonstrate that improving the education and training of management of patients with Lp(a) and advisory comments are likely to be important in changing the behaviour of specialists concerning the management of elevated Lp(a), consistent with other literature (1, 17) Hitherto, there is no consensus or guidelines on Lp(a) testing and management in Singapore or neighbouring countries. Use of electronic health system to improve testing for Lp(a) was also considered an important enabling strategy. It remains unlikely that *LPA* genetic risk score has significant clinical utility in the setting of easily measurable blood concentration of Lp(a) (1), particularly in the Asian population (22).

A limitation of this study was the small sample size, and the results may not be generalizable to all specialists or primary care doctors in Singapore. As with any survey, there may be inherent bias from selective contributions from motivated specialists. However, our study sample size represented one-sixth of the registered specialists with representations in both the public and private sectors at similar proportions to the whole nation (23), and consisted of only practicing consultants of high patient volume load, indicating that their opinions were indeed valuable.

To align with fundamental criteria for screening, the causal relationship of Lp(a) and ASCVD risk requires confirmation from cardiovascular outcome trials of highly effective Lp(a)-lowering agents, with demonstration of cost-effectiveness (3, 5). Such studies are currently underway (24). Lp(a) testing is also highly recommended by multiple clinical guidelines because Mendelian randomisation studies, large epidemiological studies and lipid apheresis studies support the causal relationship of Lp(a) and ASCVD risk, and that the diagnosis of this condition allows intensification of modifiable risk factors and overall ASCVD risk reduction (1, 5, 7, 11).

## Conclusion

We demonstrate that most cardiologists and endocrinologists in Singapore did not consistently test their patients at high risk or very high risk for ASCVD for Lp(a). This finding, compounded by the major gaps in awareness and knowledge of specialists, may explain the wide gap in detection, diagnosis, and counselling of elevated Lp(a) in patients at high and very high risk for a cardiovascular event. We identified that important actionable strategies including adequate training and facilitation of identification of at-risk patients [e.g., implementation into electronic system with interpretative comments (25), possibly pre-appointment reminders (26)] may be useful. Coordinated efforts in the hospital, primary care, nationally and internationally are necessary for implementation of proper care of individuals with elevated Lp(a), which is now recognised by many expert bodies as a public health problem. Further studies are required in other sample populations in Asia Pacific regions, specifically in lower- and middle-income countries. Finally, the value of specific educational and training strategies in improving the shortfall in care of high Lp(a) we have shown in this study, remains to be demonstrated.

## Data Availability

The original contributions presented in the study are included in the article/Supplementary Material, further inquiries can be directed to the corresponding author.

## References

[1] Kronenberg F Mora S Stroes ESG Ference BA Arsenault BJ Berglund L Dweck MR Lipoprotein(a) in atherosclerotic cardiovascular disease and aortic stenosis: a European atherosclerosis society consensus statement. Eur Heart J. (2022) 43(39):3925–46. 10.1093/eurheartj/ehac361 36036785 PMC9639807

[2] Ward NC Watts GF Bishop W Colquhoun D Hamilton-Craig C Hare DL Australian atherosclerosis society position statement on lipoprotein(a): clinical and implementation recommendations. Heart Lung Circ. (2023) 32(3):287–96. 10.1016/j.hlc.2022.11.015 36707360

[3] Loh WJ Watts GF. Detection strategies for elevated lipoprotein(a): will implementation let the genie out of the bottle? Curr Opin Endocrinol Diabetes Obes. (2023) 30(2):94–102. 10.1097/MED.0000000000000789 36468313

[4] Pearson GJ Thanassoulis G Anderson TJ. Barry AR Couture P Dayan N 2021 Canadian cardiovascular society guidelines for the management of dyslipidemia for the prevention of cardiovascular disease in adults. Can J Cardiol. (2021) 37(8):1129–50. 10.1016/j.cjca.2021.03.016 33781847

[5] Nestel P Loh WJ Ward NC Watts GF. New horizons: revival of lipoprotein (a) as a risk factor for cardiovascular disease. J Clin Endocrinol Metab. (2022) 107(11):e4281–94. 10.1210/clinem/dgac541 36108076

[6] Tsimikas S Fazio S Ferdinand KC Ginsberg HN Koschinsky ML Marcovina SM NHLBI working group recommendations to reduce lipoprotein(a)-mediated risk of cardiovascular disease and aortic stenosis. J Am Coll Cardiol. (2018) 71(2):177–92. 10.1016/j.jacc.2017.11.014 29325642 PMC5868960

[7] Koschinsky ML Bajaj A Boffa MB Dixon DL Ferdinand KC Gidding SS A focused update to the 2019 NLA scientific statement on use of lipoprotein(a) in clinical practice. J Clin Lipidol. (2024) 18(3):e308–19. 10.1016/j.jacl.2024.03.001 38565461

[8] Tsimikas S. A test in context: lipoprotein(a): diagnosis, prognosis, controversies, and emerging therapies. J Am Coll Cardiol. (2017) 69(6):692–711. 10.1016/j.jacc.2016.11.042 28183512

[9] Mach F Baigent C Catapano AL Koskinas KC Casula M Badimon L 2019 ESC/EAS guidelines for the management of dyslipidaemias: lipid modification to reduce cardiovascular risk. Eur Heart J. (2020) 41(1):111–88. 10.1093/eurheartj/ehz455 31504418

[10] Chua F Lam A Mak YH Lee ZH Dacay LM Yew JL Undiagnosed cardiovascular risk factors including elevated lipoprotein(a) in patients with ischaemic heart disease. Front Epidemiol. (2023) 3:1207752. 10.3389/fepid.2023.1207752 38455910 PMC10911051

[11] Catapano AL Daccord M Damato E Humphries SE Neely RDG Nordestgaard BG How should public health recommendations address Lp(a) measurement, a causative risk factor for cardiovascular disease (CVD)? Atherosclerosis. (2022) 349:136–43. 10.1016/j.atherosclerosis.2022.02.013 35292153

[12] Reyes-Soffer G Yeang C Michos ED Boatwright W Ballantyne CM. High lipoprotein(a): actionable strategies for risk assessment and mitigation. Am J Prev Cardiol. (2024) 18:100651. 10.1016/j.ajpc.2024.100651 38646021 PMC11031736

[13] Eidensohn Y Bhatla A Ding J Blumenthal RS Martin SS Marvel FA. Testing practices and clinical management of lipoprotein(a) levels: a 5-year retrospective analysis from the Johns Hopkins Hospital. Am J Prev Cardiol. (2024) 19:100686. 10.1016/j.ajpc.2024.100686 39070024 PMC11278112

[14] Bhatia HS Hurst S Desai P Zhu W Yeang C. Lipoprotein(a) testing trends in a large academic health system in the United States. J Am Heart Assoc. (2023) 12(18):e031255. 10.1161/JAHA.123.031255 37702041 PMC10547299

[15] Loh WJ Chang X Aw TC Phua SK Low AF Chan MY-Y Lipoprotein(a) as predictor of coronary artery disease and myocardial infarction in a multi-ethnic Asian population. Atherosclerosis. (2022) 349:160–5. 10.1016/j.atherosclerosis.2021.11.018 34887076

[16] Virani SS Koschinsky ML Maher L Mehta A Orringer CE Santos RD Global think tank on the clinical considerations and management of lipoprotein(a): the top questions and answers regarding what clinicians need to know. Prog Cardiovasc Dis. (2022) 73:32–40. 10.1016/j.pcad.2022.01.002 35063437

[17] Catapano AL. Tokgözoğlu L Banach M Gazzotti M Olmastroni E Casula M Evaluation of lipoprotein(a) in the prevention and management of atherosclerotic cardiovascular disease: a survey among the Lipid Clinics Network. Atherosclerosis. (2023) 370:5–11. 10.1016/j.atherosclerosis.2023.02.007 36894469

[18] D’Souza J Soffer DE Bajaj A. Attitudes and barriers to lipoprotein(a) testing: a survey of providers at the university of Pennsylvania health system. J Clin Lipidol. (2024) 18(4, Supplement):e487–8. 10.1016/j.jacl.2024.04.007 39289122

[19] Cicero AFG Fogacci F Giovannini M Grandi E D'Addato S Borghi C. Estimating the prevalence and characteristics of patients potentially eligible for lipoprotein(a)-lowering therapies in a real-world setting. Biomedicines. (2023) 11(12):3289. 10.3390/biomedicines11123289 38137510 PMC10741849

[20] Loh WJ Pang J Chakraborty A Ward NC Chan DC Hooper AJ Cascade testing of children and adolescents for elevated lp(a) in pedigrees with familial hypercholesterolaemia. J Clin Lipidol. (2024) 18(1):e33–7. 10.1016/j.jacl.2023.11.007 38040538

[21] Chakraborty A Chan DC Ellis KL Pang J Barnett W Woodward AM Cascade testing for elevated lipoprotein(a) in relatives of probands with high lipoprotein(a). Am J Prev Cardiol. (2022) 10:100343. 10.1016/j.ajpc.2022.100343 35517871 PMC9062205

[22] Trinder M Uddin MM Finneran P Aragam KG Natarajan P. Clinical utility of lipoprotein(a) and LPA genetic risk score in risk prediction of incident atherosclerotic cardiovascular disease. JAMA Cardiol. (2021) 6(3):287–95. 10.1001/jamacardio.2020.5398 33021622 PMC7539232

[23] SMC. Singapore Medical Council Annual Report 2022.

[24] Nordestgaard BG Langsted A. Lipoprotein(a) and cardiovascular disease. Lancet. (2024) 404(10459):1255–64. 10.1016/S0140-6736(24)01308-4 39278229

[25] Bell DA Bender R Hooper AJ McMahon J Edwards G van Bockxmeer FM Impact of interpretative commenting on lipid profiles in people at high risk of familial hypercholesterolaemia. Clin Chim Acta. (2013) 422:21–5. 10.1016/j.cca.2013.03.027 23566930

[26] Eid WE Sapp EH Conroy C Bessinger C Moody CL Yadav R Increasing provider awareness of Lp(a) testing for patients at risk for cardiovascular disease: a comparative study. Am J Prev Cardiol. (2025) 21:100895. 10.1016/j.ajpc.2024.100895 39720768 PMC11666892

